# B cells are Essential for Memory T cell Development in Germinal Centers

**DOI:** 10.33696/immunology.8.257

**Published:** 2026

**Authors:** Gongyi Zhang

**Affiliations:** 1 Department of Immunology and Genomic Medicine, National Jewish Health, Denver, CO 80206, USA; 2 Department of Immunology and Microbiology, School of Medicine, University of Colorado Anschutz Medical Campus, Aurora, CO 80216, USA

**Keywords:** B cell, T cell, Dendritic cell, Germinal center, Follicular T helper cells, Memory T cell, BCL6

## Abstract

Nature recently listed 20 major T cell milestones since the early breakthroughs of Drs. Jacques Miller and Max Cooper. However, to make the story more complete, several key discoveries may need to be mentioned. One of the major missing episodes is the discovery of the bone marrow in mice as the origin of T and B cells, revealed by Dr. Henry Claman and his colleagues. The complete absence of the critical role of B cells in the final differentiation of T cells into memory lineages could be a major oversight in modern immunology. In this short essay, I will try to summarize some of the published evidence showing that T cell differentiation into memory cells is triggered only by B cells in germinal centers after priming at infection sites for CD8 T cells and in the T cell zone for CD4 T cells in the spleen and draining lymph nodes. Based on the analysis, T cells need two steps to develop into long-lived memory cells, and a new T cell lineage model is presented.

## Introduction

A collection of major milestones in T cells was recently published in Nature (https://www.nature.com/immersive/d42859-022-00032-7/index.html) since the discoveries and characterization of two major players in adaptive immunity, T cells and B cells, by Drs. Jacques Miller [1], Max Cooper [2] in the 1960s. However, the discovery of the mammalian (mouse) bone marrow as the primordial lymphoid lymphatic cell reservoir (including T cells, B cells, and other immune cells) by Claman and his colleagues actually opened up modern immunology [3]. Literally, if not all, at least most of the milestones listed were produced from the mouse model. However, this essay only focuses on one of the two most critical sides of the relationship between B cells and T cells: how B cells help T cells to differentiate into memory cells, since how T cells help B cells was one of the main themes of the listed milestones. An overwhelming bias of research in the field of immunology is how T cells determine the functional proliferation and differentiation of B cells in animals. It took almost half a century for people to elucidate the detailed underlying mechanism of how T cells help B cells in the germinal centers (GCs) of the spleen and local lymph nodes through a special lineage of T cells, the follicular T helper cells (Tfh) [4–6]. Due to the lack of a B cell-specific maturation organ or apparatus like the Thymus for T cells, how B cells mutually help T cells (T cell proliferation and differentiation) has been overlooked or severely underappreciated in the field of immunology, although groundbreaking discoveries were made even before people revealed how Tfh cells guide the maturation process of B cells in GCs.

## B cells were Found to be Antigen-Presenting Cells

Marrack and Kappler were pioneers in showing that B cells express both Ir (MHC-II) [7] and MHC-I [8] and that hybridoma B cells present antigens to hybridoma T cells [9,10], while Chestnut and Grey showed that normal B cells present antigens to T cells as efficiently as to that of macrophages at the same time [11,12].

## Depletion of B cells Impairs T cell Proliferation in Spleen and Lymph Nodes

Ron and Segal were the first to report that B cell depletion by anti-μ antibody treatment in mice results in a defect in antigen-reactive T-cell proliferation [13,14]. Segal also demonstrated that splenic B cells function as antigen-presenting cells to prime T cells and convert T cells into effector T cells [15]. Lanzavecchia was the first to show that antigens taken up by B cells are processed within B cells and subsequently presented by B cells to T cells via the MHC molecules [16]. This was later confirmed by Janeway and Ron, who demonstrated that B cells are the antigen-presenting cells required to initiate T cells in the local lymph nodes [17,18]. Kupfer directly observed how B cells present antigens to T cells using immunofluorescence microscopy [19]. Overall, the essential role of B cells as antigen-presenting cells to drive T cell activation in the spleen and lymph nodes was established in the 1980s.

## T cells Fail to Differentiate into Memory T cells without B cells

In the early 1990s, it was reported that a continuous supply of antigens was necessary or essential for the generation of both memory T helper cells and memory cytotoxic T cells [20,21]. It was shown that B-cell depletion leads to the generation of short-lived CD8 memory T cells as well as exhausted CD8 T cells. Shortly thereafter, it was reported that depletion of B cells could lead to the failure to generate not only memory CD8 T cells but also memory CD4 T cells, conferring exhausted characteristics to both CD4 and CD8 T cells in μMT/μMT mice [22]. Shortly thereafter, it was again confirmed that B cells play a critical role in the development of CD4 memory T cells [23,24]. These results demonstrated that B cells are essential for the development of long-lasting memory T cells.

## Germinal Centers are the Place Where B cells Help T cells to Differentiate into Memory T cells

An emerging question is where B cells present antigens to T cells to help T cell differentiation. A fundamental discovery from the BCL-6-deficient mice showed that deletion of BCL-6 not only leads to the complete loss of germinal center formation but also to the failure of B cell and T cell differentiation into memory populations [25,26]. Another excellent finding from Jenkins’ group is that antigen-specific T cells migrate to the B cell zone and interact with B cells to form germinal centers shortly after antigen exposure [27]. This suggests that GCs are the main, if not the only, site for B cells to communicate with T cells. Tokuhisa’s group later showed that BCL-6 is not only essential for the development of CD8 memory T cells [28,29] but also for the development of the CD4 memory T cells [30]. A later report from Jenkins’ group showed that BCL-6 is essential for the development of all memory T cells [31], further supporting that GCs are the primary site for T cell differentiation based on the only GC-expressing feature of BCL-6. Later, follicular T cells were shown to directly support B cell differentiation in the GCs [4–6]. These results demonstrate that B cells, as the only T cell antigen-presenting cells in the GCs, are essential for memory T cell formation and that T cells promote the B cell high-affinity maturation through somatic hypermutation in a mutually beneficial scenario (Figure 1). Another surprising piece of evidence in support of this speculation is that even a single B cell clone expressing a specific antibody to a lymphocytic choriomeningitis virus (LCMV) antigen on the surface of B cell-transgenic mice on top of B cell-deficient mice could lead to normal development and differentiation of CD4 memory T cells [32].

## Dendritic Cells Prime T cells in the T cell Zone, While B cells Take Over after Priming

A common view in the immunology field is that dendritic cells (DCs) are the dominant antigen-presenting cells for T cells to proliferate and differentiate into both effector T cells and memory T cells. A seminal discovery from Jenkins’ group showed that DCs are required to prime CD4 T cells in the lymph nodes, but after priming by DCs, B cells take over from DCs to become the dominant, if not sole, antigen-presenting cells in the lymph nodes [27,33]. This suggests that DCs are only required for early CD4 T cell priming in the T cell zone of the lymph nodes during pathogen attack, while B cells are the actual players to support T cell differentiation in GCs.

## B cells are Essential for Proper T cell Differentiation in Humans

Interestingly, when anti-CD20 antibodies became therapeutic agents for the treatment of cancer and autoimmune diseases, it was found that patients treated with anti-CD20 antibodies became vulnerable to viral [34] and bacterial [35] infections with defects in memory T cells. Furthermore, patients with B-cell depletion were unable to mount normal humoral and cellular immune responses following SARS-CoV-2 vaccination [36–38]. A more striking observation was that anti-CD20-treated patients failed to clear SARS-CoV-2 [39]. The intriguing fact is that SARS-CoV-2-specific effector and memory T cells are properly produced, but without normal clearance capacity similar to that of exhausted T cells [39]. These results strongly support the concept that T cells without B cells could not properly develop into effector or memory T cells.

## A Two-Step Process, First Primed by Infected Cells (for CD8 T cells) or DCs (for CD4 T cells) and Second Activated by B cells, is Needed for Memory T cell Differentiation

Based on the above analysis of all published results, we can confidently conclude that there are two steps for CD4 T cell activation and differentiation. First, dendritic cells will present antigens to T cells and prime CD4 T cells in the T cell zone. However, CD8 T cells are usually primed at the site of infection and migrate to the T cell zone via the lymphatic circulation system (detail will be analyzed in another essay). Second, both primed CD4 T and CD8 T cells will migrate to the B cell zone and work together with B cells (Figure 2). CD4 T cells will help B cell maturation in germinal centers. Conversely, B cells are the major antigen-presenting cells that drive the T cell maturation process, which most likely occurs in the germinal centers of the spleen and local lymph nodes. It is likely that the feedback signals from germinal center B cells during the matching or pairing between B and T cells in the light zone of GCs trigger T cell activation (second time) and subsequent differentiation into memory T cells (Figure 1), suggesting that these GC-experienced T cells give rise to memory T cells. Without the help of B cells, T cells either remaining in the T cell zone or migrating into the B cell zone after priming will eventually differentiate into exhausted T cells or undergo apoptotic cell death after the resolution of infection. Furthermore, it may remind us not only that current developmental pathways or lineage classifications of memory T cells have caveats but also that current markers assigned to define different types of effector or memory T cells are questionable. Most importantly, it suggests that both follicular CD4 and CD8 cells are halfway along their journey to develop into memory T cells starting from the primed stage. After a second match with germinal center B cells, all these successfully B cell-matched T cells will differentiate into long-lived memory T cells, either memory CD8 or memory CD4 T cells. In this regard, we conclude that germinal center follicular T cells are the essential intermediate T cell lineage for the final long-lived memory T cells for both CD4 T cells (Figure 3) and CD8 T cells (not shown, similar to the CD4 T cell pathway, but CD8 T cells are first primed at infection sites by infected cells or macrophages, but not DC cells in the T cell zone).

## Figures and Tables

**Figure 1.**
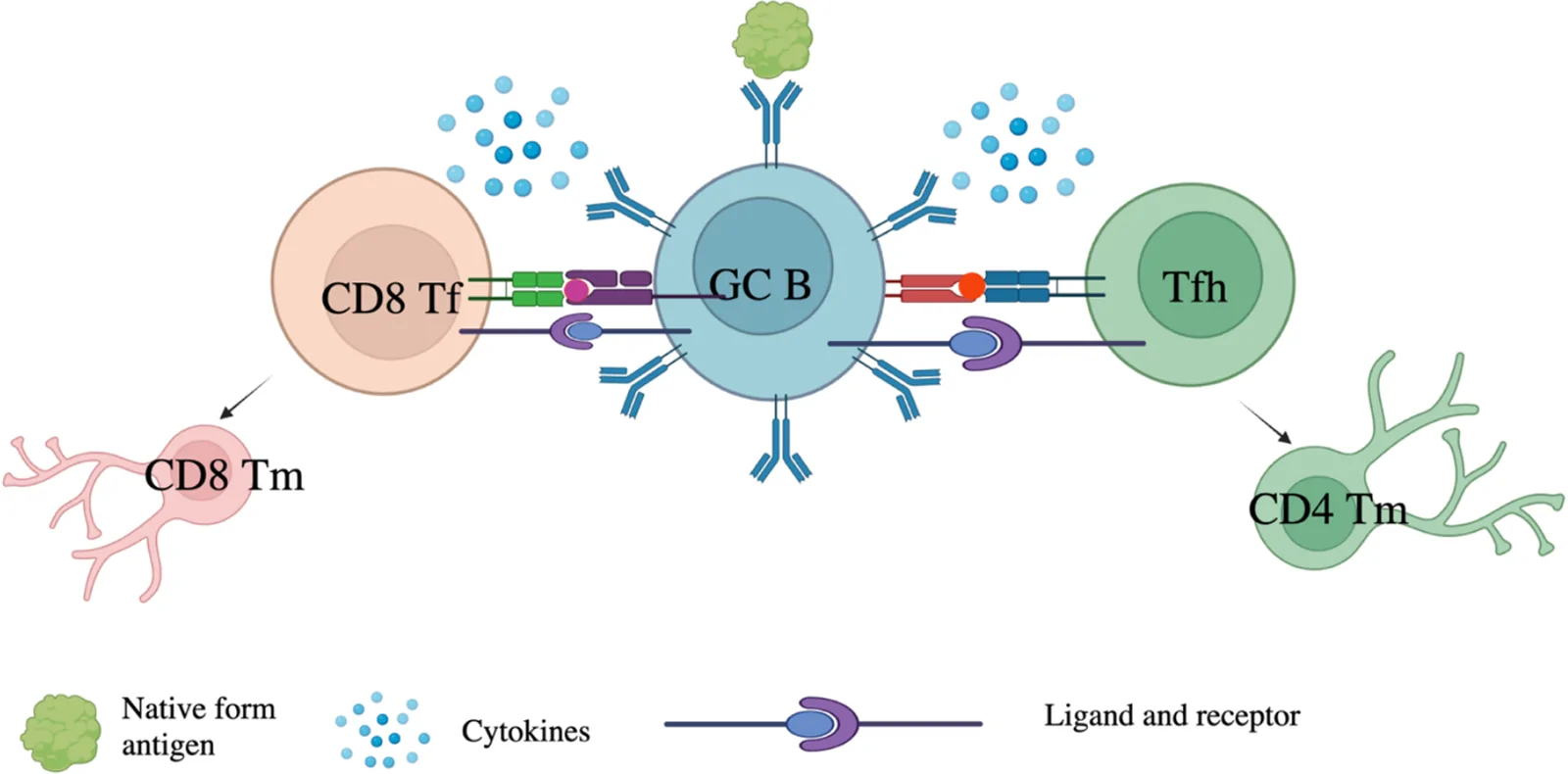
Germinal center B cells are essential for T cells to differentiate into long-lasting memory T cells. A successful pairing between CD4 Tf cells/CD8 Tf cells and B cells triggers the differentiation of Tfh cells/CD8 Tf cells into memory CD4 T cells/CD8 T cells.

**Figure 2.**
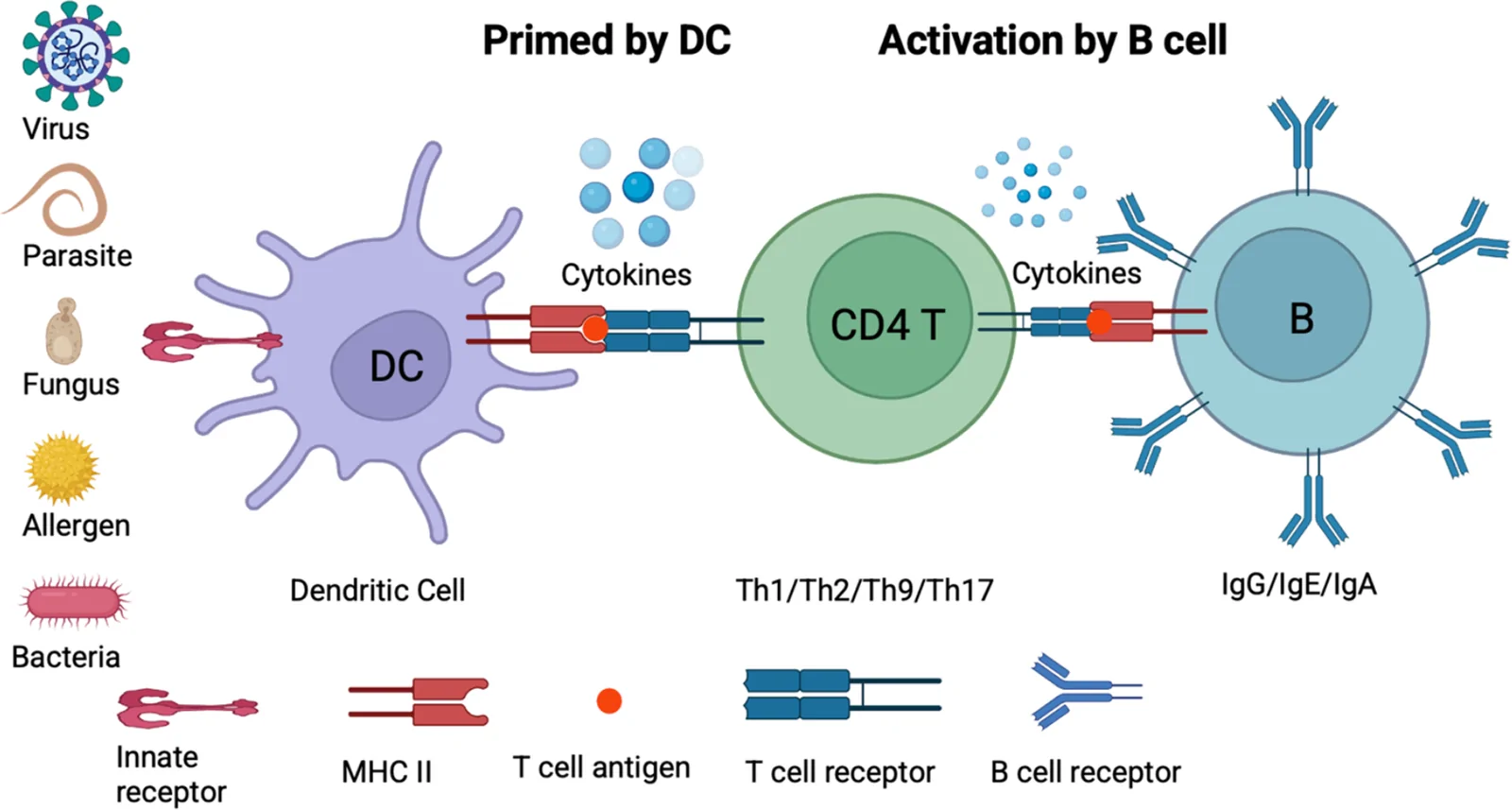
Two steps are needed for CD4 T cells to differentiate into long-lived memory CD4 T cells. Step one, T cells are primed by dendritic cells in T cell zone. Step two: A portion of primed T cells migrate into B cell zone and are activated by B cells in the germinal centers.

**Figure 3.**
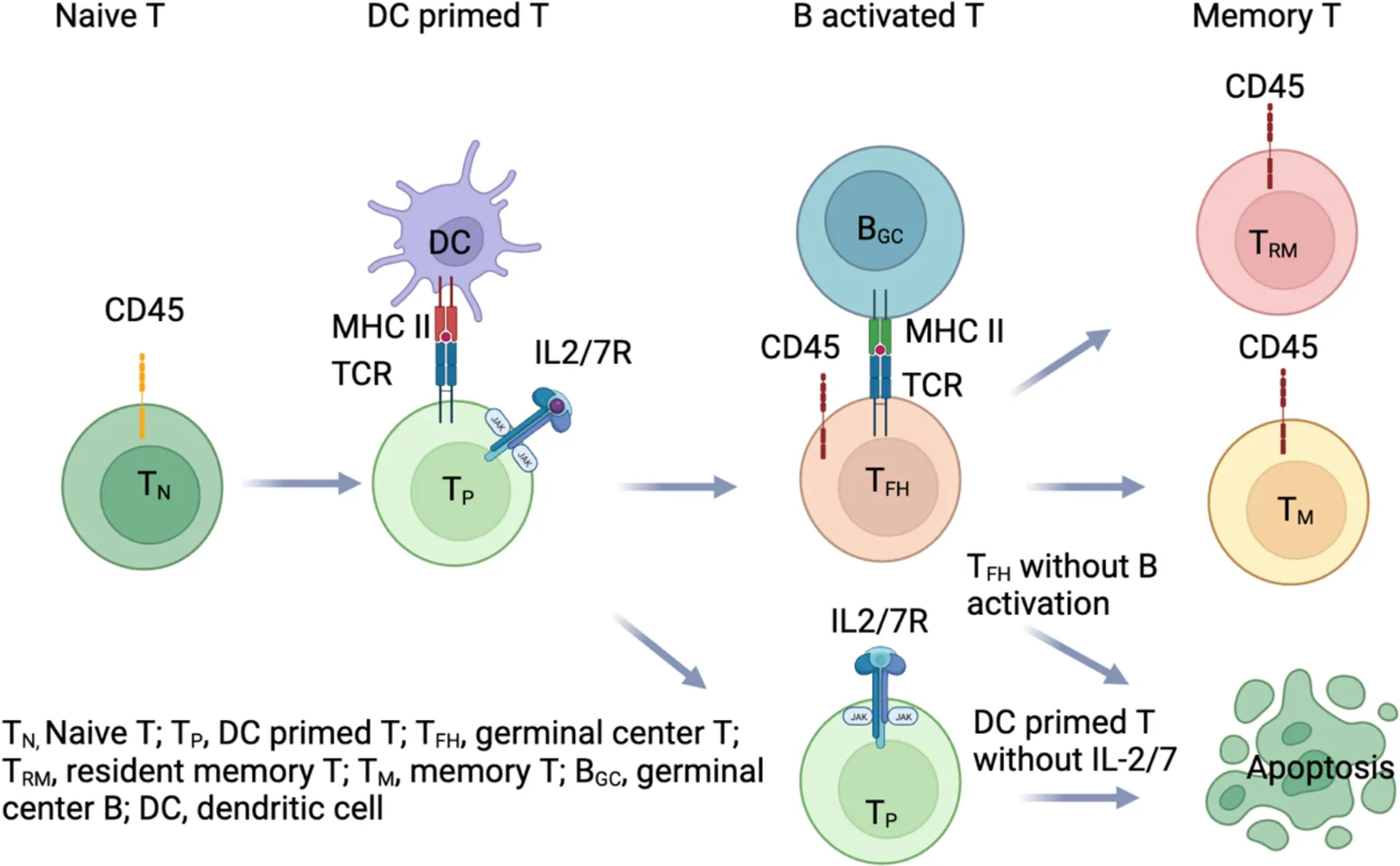
The potential CD4 T cell lineage model. Naïve CD4 T cells are primed by dendritic cells in T cell zone. A portion of primed T cells will migrate into follicular B cell zone and form germinal center with B cells. Germinal center T cells will guide B cell maturation. At the same time, T cells will be activated by B cells and eventually differentiate into memory T cells after resolution of infection and desolvation of germinal centers.
